# Mutation of an Arabidopsis NatB N-Alpha-Terminal Acetylation Complex Component Causes Pleiotropic Developmental Defects

**DOI:** 10.1371/journal.pone.0080697

**Published:** 2013-11-14

**Authors:** Almudena Ferrández-Ayela, Rosa Micol-Ponce, Ana Belén Sánchez-García, María Magdalena Alonso-Peral, José Luis Micol, María Rosa Ponce

**Affiliations:** Instituto de Bioingeniería, Universidad Miguel Hernández, Campus de Elche, Elche, Spain; Centro de Investigación en Medicina Aplicada (CIMA), Spain

## Abstract

N-α-terminal acetylation is one of the most common, but least understood modifications of eukaryotic proteins. Although a high degree of conservation exists between the N-α-terminal acetylomes of plants and animals, very little information is available on this modification in plants. In yeast and humans, N-α-acetyltransferase complexes include a single catalytic subunit and one or two auxiliary subunits. Here, we report the positional cloning of *TRANSCURVATA2* (*TCU2*), which encodes the auxiliary subunit of the NatB N-α-acetyltransferase complex in Arabidopsis. The phenotypes of loss-of-function *tcu2* alleles indicate that NatB complex activity is required for flowering time regulation and for leaf, inflorescence, flower, fruit and embryonic development. In double mutants, *tcu2* alleles synergistically interact with alleles of *ARGONAUTE10*, which encodes a component of the microRNA machinery. In summary, NatB-mediated N-α-terminal acetylation of proteins is pleiotropically required for Arabidopsis development and seems to be functionally related to the microRNA pathway.

## Introduction

One of the most common modifications of eukaryotic proteins is N-α-acetylation of the amino terminus, which is found in 50–70% of yeast proteins and 70–90% of human proteins, but occurs only rarely in prokaryotic proteins (reviewed in [1]). N-α-acetylation occurs in the cytoplasm and is carried out by N-α-acetyltransferases, which transfer an acetyl group from acetyl-CoA to the α-amino group of the first amino acid of a protein. Unlike other protein modifications, N-α-terminal acetylation is irreversible and, although it was first described over 50 years ago [2], little is known about its function.

In yeast and mammals, N-α-terminal acetylation involves several complexes, termed NatA to NatF, which exhibit substrate specificity (reviewed in [1]). The NatA, NatB and NatC complexes contain a catalytic subunit and one or two auxiliary, non-catalytic subunits. The Nat3 (N-acetyltransferase 3) and Mdm20 (mitochondrial morphology and distribution 20) subunits of the yeast and human NatB complex cosediment with ribosomes, which suggests that they act on nascent polypeptides [3]–[5]. A recently revised nomenclature for N-terminal acetyltransferases [6] renamed the Nat3 and Mdm20 proteins as Naa20 and Naa25, respectively. The human NatB complex seems to be required for cell cycle progression [5]. The Ard1 (Naa10) catalytic subunit of the human NatA complex has been isolated in cytoplasmic and nuclear fractions, which suggests additional functions for Ard1, other than cotranslational N-α-terminal acetylation [7]. Recent work showed that the X-linked Ogden syndrome is caused by a specific missense mutation affecting the Naa10 catalytic subunit of the human NatA complex [8].

Several studies in yeast and mammals have shown that N-α-terminal acetylation has diverse molecular functions including inhibition of protein translocation to endoplasmic reticulum [9], regulation of protein degradation [10] and mediation of protein interactions [11]. It has been believed for many years that N-α-terminal acetylation protects proteins from degradation [12], [13]. In fact, proteins with N-α-terminal acetylation are more stable *in vivo* than non-acetylated proteins [14]. This could be due to ubiquitination, which involves direct conjugation of ubiquitin to the N-terminal amino acid and promotes the subsequent degradation of ubiquitinated proteins [15]. Thus, acetylation of the amino terminus would prevent ubiquitination and stabilize proteins [16]. This occurs, for example, with the p16 and p14/p19ARF tumor suppressor proteins in human and mouse cells [15], [17], [18]. However, an acetylated amino terminus did not affect the destabilization of a protein by ubiquitin-independent mechanisms [19]. In fact, it has been proposed that, in some cases, acetylated amino terminal sequences may act as signals for degradation [10], [20]. At least eight yeast proteins that are acetylated at their amino termini are recognized by the Doa10 ubiquitin ligase [10], [16], [21]. It has been shown recently in yeast that NatB-mediated acetylation does not alter the stability of proteins [22]. Although these observations suggest opposite roles for N-α-terminal acetylation, several mechanisms could coexist, each acting on specific groups of proteins under certain conditions.

N-α-terminal acetylation has different functions in different proteins, as shown by studies performed in yeast [16]: actin and tropomyosin activity requires NatB-mediated N-α-terminal acetylation [3], [23], [24]; Tfs1p (Twenty-five suppressor) lipid binding protein requires N-terminal acetylation by NatB to inhibit carboxypeptidase Y [25]; delivery of the Arl3p (Arf-related protein 3) and Grh1p (GRASP65 protein homologue 1) GTPases to the Golgi apparatus requires NatC-mediated acetylation [26]–[28]; association of Trm1p-II (Transcriptional regulation of methanol induction 1 protein II) with the inner nuclear membrane also requires acetylation [29]; and Sir3p (Silent information regulator 3 protein) and Orc1p (Origin recognition complex 1 protein) activities in gene silencing require acetylation by NatA [30], [31]. However, the mechanisms by which acetylation affects protein function remain unclear. For example, a study of several NatB substrates led to the conclusion that the absence of acetylation has no apparent impact on their subcellular localization [32].

There is a dearth of information on the activity of Nat complexes in plants. Mutant phenotypes caused by genes encoding components of the Nat complexes have been identified for only one gene, that encoding the MAK3 (NAA30) catalytic subunit of NatC. The *atmak3-1* mutation was isolated in a search for insertional mutants with reduced performance of photosystem II and exhibits a smaller rosette, paler leaves and less thylakoidal multiprotein complexes than wild type [33]. The Mak3p (Naa30p), Mak10p (Naa35p) and Mak31p (Naa38p) components of yeast NatC are essential [34], but null alleles of the putative Arabidopsis ortholog of yeast *MAK10* do not cause any visible mutant phenotype. In Arabidopsis, NatC-mediated N-α-terminal acetylation of certain chloroplast protein precursors seems to be necessary for the accumulation of mature proteins in this organelle [33]. A recent proteome-wide mass spectrometry analysis that allowed enrichment and selection of N-terminal peptides from Arabidopsis and human samples identified hundreds of N-α-acetylated proteins; the results indicated a high degree of convergence between the plant and animal kingdoms in both components and substrates of the N-α-acetylation machinery. An unknown mechanism seems to be responsible for N-α-acetylation (after removal of the transit peptide) of a high proportion of chloroplast proteins encoded by Arabidopsis nuclear genes [35].

Here, we report the positional cloning and characterization of Arabidopsis *TRANSCURVATA2* (*TCU2*), which encodes the auxiliary subunit of the NatB complex. The pleiotropic phenotypes caused by loss of function of *TCU2* indicate that NatB-mediated N-α-terminal acetylation plays a role in leaf flatness, flowering time, inflorescence structure, flower size, silique structure and size, and embryonic development. In addition, genetic interactions between *TCU2* alleles and alleles of *ARGONAUTE10* (*AGO10*) suggest an unexpected link between N-α-terminal acetylation and the microRNA pathway.

## Results

### Morphological and histological phenotypes of the P14 6.3 mutant line

**Figure 1:**
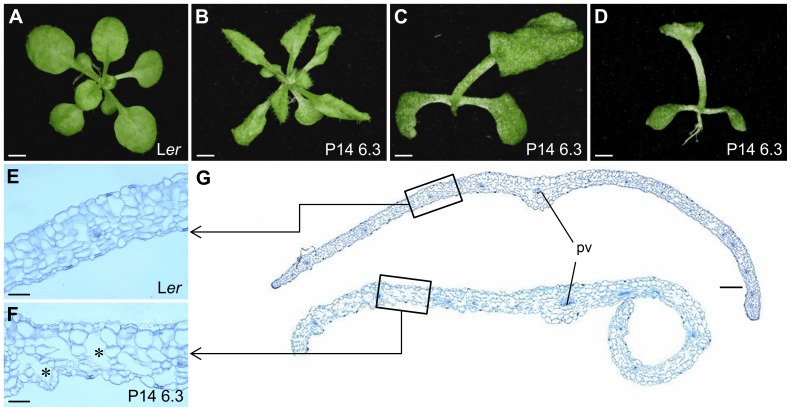
Leaf morphology and histology in the P14 6.3 line. (A, B) Rosettes of (A) L*er* and (B) P14 6.3 plants. (C) Top and (D) lateral views of a P14 6.3 plant showing fusion of the first pair of vegetative leaves. (E, F) Transverse sections of the central region of the lamina, midway between the leaf margin and the primary vein of (E) L*er* and (F) P14 6.3 third-node leaves. Asterisks indicate large intercellular air spaces. (G) Margin to margin transverse sections of third-node leaves. pv: primary vein. Pictures were taken (A–D) 22 das and (E–G) 21 das. Scale bars: (A, B, D) 2 mm, (C) 1 mm, (E, F) 50 µm and (G) 200 µm.

A screen for ethylmethane sulfonate (EMS)-induced leaf mutants in a L*er* background, conducted in the laboratory of J.L. Micol [36], identified two lines (P14 6.3 and P14 20.1) with indistinguishable phenotypes, vegetative leaves that bend down obliquely to the primary vein. We tested for monogenic inheritance in the F_2_ of their backcrosses to L*er* using a chi-square test with the Yates correction for one degree of freedom. These mutant lines exhibit monogenic recessive inheritance, as shown by the 3∶1 phenotypic segregation ratios found for P14 6.3 (78 wild-type plants: 15 mutant plants; chi-square = 3.44) and P14 20.1 (79∶19; chi-square = 1.36). Since these non-complementing lines had been isolated from the same parental group (see Methods), they were considered likely to be identical, and only the P14 6.3 line (Figure 1B) was further studied.

The vegetative leaves of P14 6.3 are reticulated with protuberant veins that are greener than the interveinal tissues. Around 10% of P14 6.3 plants (Figure 1C, D) showed only one large leaf with a duplicated primary vein, fusion of the first two leaves, or a single, trumpet-shaped leaf apparently abaxialized; these aberrant seedlings are viable and develop into fertile plants. This phenotype, which is likely a consequence of perturbation of shoot apical meristem function, was evident in around 10% of the progeny resulting from selfing of any P14 6.3 plant, regardless of the parental phenotype. Plants with this phenotype were not examined in the phenotypic analyses described below.

Vegetative leaves are smaller in P14 6.3 than in wild type, except for the first pair, which are similar to those of L*er*. To study their morphology and histology, first- and third-node P14 6.3 leaves were collected 21 days after stratification (das) and cleared with chloral hydrate. Dark-field micrographs were used to draw venation pattern diagrams (Figure S1), which were subjected to morphometric analysis. Only small differences in venation pattern were found with the wild type, except for the duplicated primary vein shown by the fused first pair of leaves occasionally seen in P14 6.3 seedlings.

**Figure 2:**
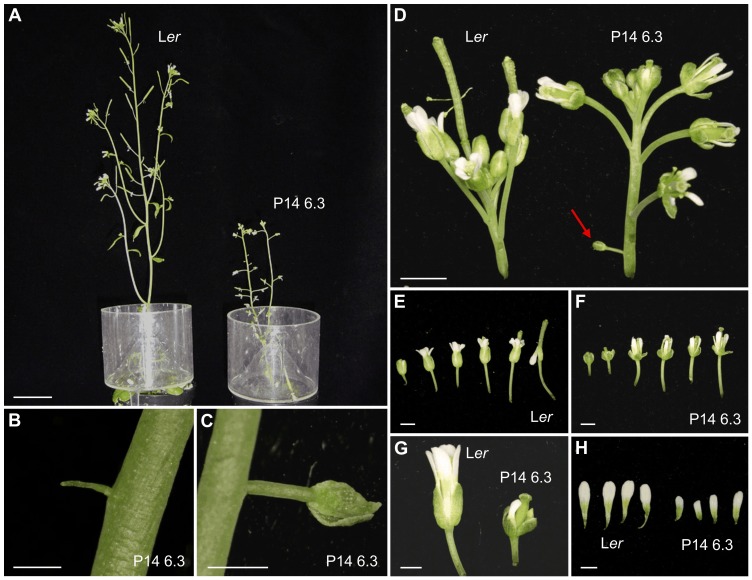
Inflorescence structure and floral morphology in P14 6.3. (A) Lateral view of L*er* and P14 6.3 inflorescences. (B, C) Details of abnormal structures observed in P14 6.3 stems: (B) a filamentous bulge, and (C) an aborted lateral flower. (D) Inflorescence apex in L*er* and P14 6.3 plants. The red arrow indicates an aborted lateral flower. (E, F) Flowers of the inflorescence apex from (E) L*er* and (F) P14 6.3. (G, H) Different sizes of (G) flowers from the inflorescence apex and (H) petals from L*er* and P14 6.3. Pictures were taken 35 das. Scale bars: (A) 2 cm, (B, C) 0.5 mm, (D–F) 2 mm and (G, H) 1 mm.

**Figure 3:**
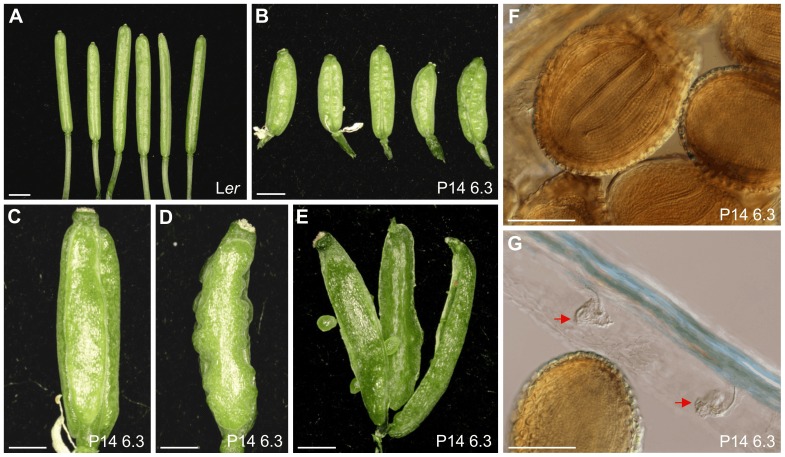
Silique morphology in P14 6.3. (A, B) Siliques from (A) L*er* and (B) P14 6.3 plants. (C–E) Three valves with a rough surface were shown in most P14 6.3 siliques: (C) front and (D) lateral views of closed siliques and (E) a silique with longitudinally open septa. (F) Seeds containing mature embryos and (G) aborted or unfertilized ovules (red arrows) in dissected P14 6.3 siliques. Pictures were taken 35 das. Scale bars: (A, B) 2 mm, (C–E) 1 mm and (F, G) 200 µm.

Optical microscopy of leaf cross-sections (Figure 1E-G) showed that there is more heterogeneity in mesophyll cell and airspace sizes in P14 6.3 than in wild type. No differences with the wild type were observed in root apex structure, root hair number and root length, although root hairs are longer in P14 6.3 than in L*er*. In P14 6.3, the stem is shorter than wild type (Figure 2A), is fasciated and winding, and has some lateral filiform bumps and aborted flowers (Figure 2B, C). In L*er*, flowers appear clustered and separate from each other when siliques begin to elongate, but in P14 6.3 they separate from the cluster before silique formation (Figure 2D). Floral organs of P14 6.3 are shorter, particularly the petals (Figure 2E-H). Mutant siliques are short, thick and rugged compared to those of L*er*, contain many aborted or unfertilized ovules and almost all exhibit three or even four valves instead of the two usually seen in wild type (Figure 3).

### Positional cloning of the *TCU2* gene

**Figure 4:**
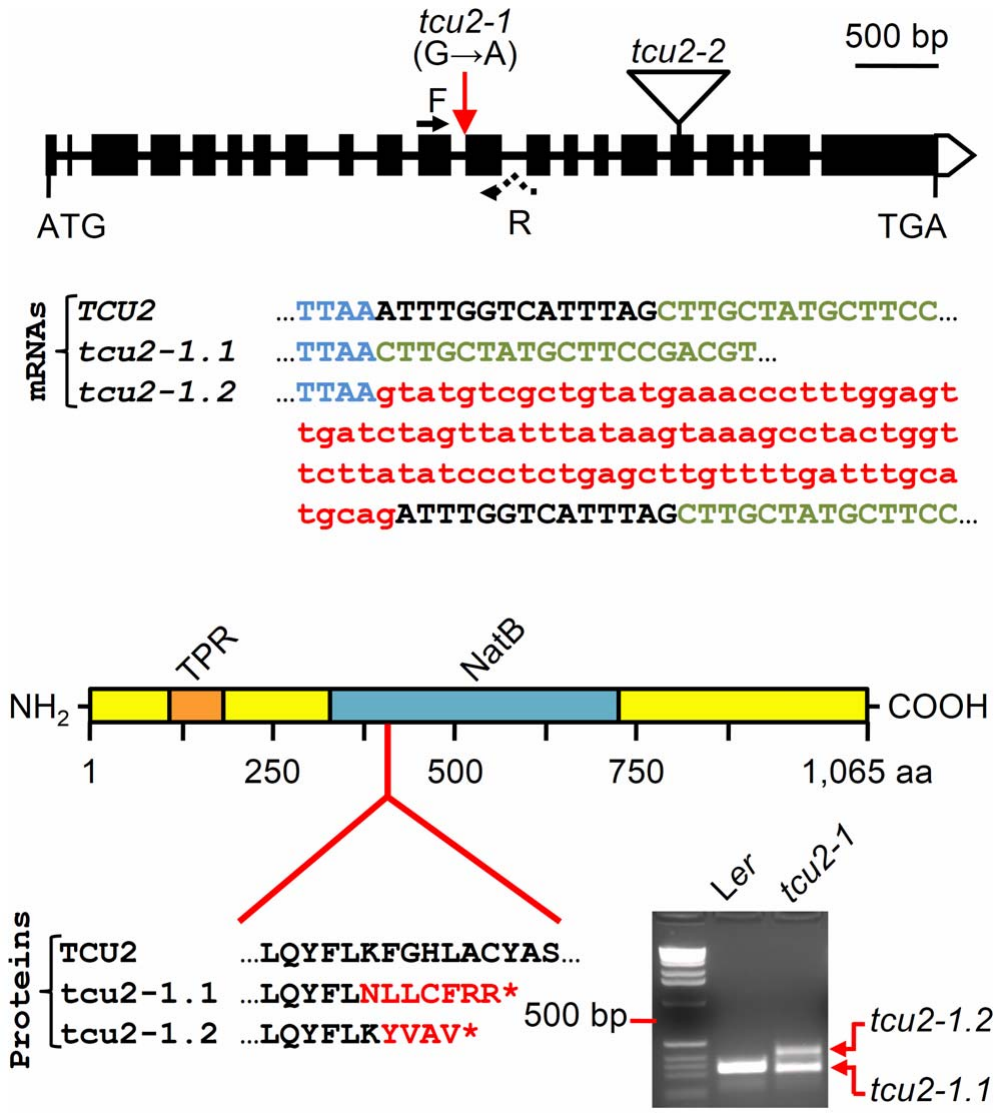
Structure of the *TCU2* gene, its mutant alleles and their effects on the TCU2 protein. Blue and green capital letters represent nucleotides of exons eleven and twelve, respectively, of the *TCU2* gene, which are present in *TCU2* wild-type mRNA and the *tcu2-1.1* and *tcu2-1.2* mutant mRNA variants. Red lowercase letters highlight the intron that is not removed from *tcu2-1.2* during splicing; black capital letters indicate the nucleotides from the 5′ end of wild type exon twelve that are removed from *tcu2-1.1* during splicing. The TCU2 protein contains several tetratricopeptide repetitions (TPR; residues 89 to 181) and a NatB domain (residues 365 to 725). The mutant proteins have 436 (tcu2-1.1) and 434 (tcu2-1.2) amino acids. A picture of a 3%-agarose gel stained with ethidium bromide is shown at the lower right corner, which shows the PCR amplification products obtained from reverse transcribed total mRNA extracted from L*er* and P14 6.3 whole plants.

We termed the causal mutation for the phenotype of the P14 6.3 line *transcurvata2-1* (*tcu2-1*). Low-resolution mapping of the *tcu2-1* mutation had already been carried out by linkage analysis [37]. We developed new molecular markers (Table S1), most of them based on the small insertions or deletions (In/Del) described in the Monsanto database (http://www.arabidopsis.org/browse/Cereon/index.jsp) and used them for the analysis of a mapping population of 1,137 F_2_ plants derived from a P14 6.3×Col-0 cross. This allowed us to define a 176-kb candidate interval encompassing 52 annotated genes (Figure S2). To identify *TCU2*, we first obtained 81 lines carrying T-DNA insertions in genes in the candidate interval; however, none of the lines exhibited the same mutant phenotype as *tcu2-1*. However, sequencing of 28 of the candidate genes in the *TCU2* region revealed a G→A transition in the last nucleotide of the eleventh intron of At5g58450. This mutation damages a splicing acceptor site, generating two aberrant mRNA variants, one of which (*tcu2-1.2*) retains intron eleven of At5g58450, while the other (*tcu2-1.1*) lacks the first 14 nucleotides of exon twelve. We demonstrated these alterations by sequencing PCR amplification products obtained from reverse transcribed *tcu2-1* mRNA (Figure 4).

### P14 6.3 is a *tcu2-1 zll-2* double mutant

**Figure 5:**
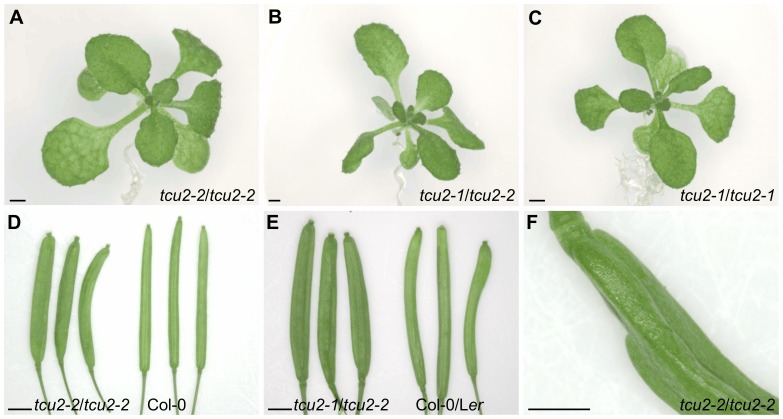
Some phenotypic traits of the *tcu2-2* mutant. (A–C) Rosettes of (A) *tcu2-1/tcu2-1*, (B) *tcu2-2/tcu2-2* and (C) *tcu2-1/tcu2-2* plants. (D) Siliques from *tcu2-2/tcu2-2* (left) and Col-0 (right) plants. (E) *tcu2-1/tcu2-2* (left) and wild-type sibling (right) siliques. Only three-valved siliques are shown from *tcu2-2/tcu2-2* and *tcu2-1/tcu2-2* in D and E; the wild-type siliques shown have two valves. (F) Detail of a *tcu2-2/tcu2-2* silique with four valves. Pictures were taken (A, C) 21 das, (B) 18 das and (D-F) 50 das. Scale bars: (A–E) 2 mm and (F) 1 mm.

We found several publicly available lines carrying T-DNA insertions in At5g58450 in the Col-0 genetic background, but only one line (GK_819_A05) exhibited a mutant phenotype. The corresponding allele of *TCU2* was termed *tcu2-2* (Figure 4). As observed in P14 6.3, plants homozygous for the *tcu2-2* allele showed reticulated leaves. However, the strong leaf folding that characterizes P14 6.3 plants was mild in *tcu2-2* plants. Both reticulation and weak folding were similar in the leaves of *tcu2-2* homozygous plants and the *tcu2-1/tcu2-2* heterozygous F_1_ progeny of a P14 6.3×*tcu2-2* cross (Figure 5A, B). We concluded that these two lines are allelic and that some phenotypic traits of *tcu2-1* are influenced by a recessive modifier present in the L*er* background of P14 6.3 but absent in the Col-0 background of *tcu2-2*.

Four phenotypic traits that the P14 6.3 and *tcu2-2* lines do not share are: fusion of the first two leaves (in about 10% of P14 6.3 plants but never seen in *tcu2-2*); reduced silique length (only in P14 6.3); three or more valves (in almost all P14 6.3 siliques but only in some *tcu2-2* siliques) and reduced plant stature (small in P14 6.3 but similar to wild type in *tcu2-2*). The first three of these traits can be caused in a L*er* genetic background by loss-of function alleles of *AGO10* (*ARGONAUTE10*; also called *PINHEAD* [*PNH*] and *ZWILLE* [*ZLL*]), a known component of the microRNA (miRNA) machinery [38]–[40]. *AGO10* and *TCU2* are linked at a distance of about 30 cM. Taken together, these observations prompted us to obtain a mutant carrying the *pnh-2* loss-of-function allele of *AGO10*, in a L*er* genetic background. The *pnh-2* mutant exhibited the expected, previously published phenotype [41], [42] under our growth conditions (Figure S3): around 10% of the *pnh-2* plants showed fusion of the first two leaves, and all plants exhibited small flowers, which developed into thick siliques, most of which had three valves, containing some aborted or unfertilized ovules. These phenotypic traits were less severe in *pnh-2* than in P14 6.3.

**Figure 6:**
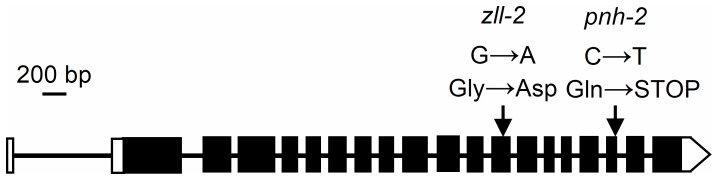
Structure of the *AGO10* gene and its alleles studied in this work. Exons and introns are shown as black rectangles and lines, respectively. White boxes represent the 5′ and 3′ untranslated regions. Arrows point to the position of the *zll-2* and *pnh-2* point mutations.

Sequencing of *AGO10* in the P14 6.3 and P14 20.1 mutant lines and their wild type L*er* revealed a G→A transition in its exon twelve, which is predicted to cause a Gly707→Asp change in the AGO10 protein (Figure 6). Since an identical mutation has already been published and named *zll-2* [43], we decided to use this symbol for the mutation found in *AGO10* of P14 6.3 and P14 20.1. This result revealed that the latter lines carry two mutations: *tcu2-1* in *TCU2* and *zll-2* in *AGO10*. Next-generation, whole genome sequencing of P14 6.3 DNA confirmed the presence of both *tcu2-1* and *zll-2* homozygous mutations, as well as the absence of any other G→A or C→T homozygous mutations (those typically caused by EMS) affecting either exons or splicing donor or acceptor signals in the genes encompassed by the 176-kb interval candidate delimited by linkage analysis (Figure S2). In addition, we obtained *tcu2-2 pnh-2*, a different double mutant combination of loss-of-function alleles of *TCU2* and *AGO10*, whose phenotype was very similar to that of P14 6.3, the only exception being plant height, which was higher in *tcu2-2 pnh-2* than in P14 6.3 (see below).

To study their individual phenotypic effects, we segregated the *tcu2-1* and *zll-2* mutations in the F_2_ progeny of a cross of the *tcu2-1 zll-2* double mutant to L*er*. The *tcu2-1* single mutant exhibited leaf reticulation and mild leaf folding (Figure 5C), and only a few three-valved siliques (see below). By contrast, the *zll-2* single mutant showed fusion of the first two leaves in about 10% of its seedlings and most of its siliques were three-valved. Flowering was earlier in the *tcu2-1 zll-2* double mutant (22.64±0.21 das; n = 91) than in L*er* (24.98±0.28 das; n = 90) (Figure S4). Early bolting is clearly caused by the *tcu2-1* mutation, since flowering time was 22.8±0.57 das (n = 86) in the *tcu2-1* single mutant and 24.05±0.04 das (n = 91) in the *zll-2* single mutant. Because of their early flowering, adult *tcu2-1* and *tcu2-1 zll-2* plants had fewer vegetative leaves than wild type.

Only a few thick siliques with three or four valves (Figure 5D, E) were observed in heterozygous *tcu2-1/tcu2-2*, homozygous *tcu2-2* (siliques with 3 or 4 valves/all siliques = 0.02±0.03; n = 5 plants; 55 das), or homozygous *tcu2-1* (0.13±0.08) plants, but this phenotype was observed in an increased (ten-fold) proportion compared with their respective wild types (Col-0, 0.002±0.004; L*er*, 0.01±0.01). The structure of the apical region of the inflorescence was also similar in the *tcu2-1* and *tcu2-2* single mutants and in the *tcu2-1 zll-2* double mutant.

### *TCU2* encodes the auxiliary subunit of the NatB complex

The *TCU2* gene is annotated to encode a protein of 1,065 aa and 12.15 kDa (Figure 4) containing several tetratricopeptide repetitions, which are assumed to confer a helical structure and to mediate binding to other proteins. TCU2 also contains a domain with similarity to Mdm20p (Naa25p), the auxiliary subunit of the N-α-terminal acetyltransferase B (NatB) complex of organisms as diverse as mammals and yeast, which participates in the N-α-terminal acetylation of proteins.

Three N-α-terminal acetyltransferase complexes, NatA, NatB and NatC, have been characterized in *Saccharomyces cerevisiae*, each of which includes a catalytic subunit and one or more auxiliary subunits [44]. Yeast Mdm20p is the best known auxiliary, non-catalytic subunit of the NatB complex, which N-α-terminally acetylates proteins having Met-Glu, Met-Asp or Met-Asn residues at the amino terminus [3], [44]. Like Mdm20p, TCU2 contains a TPR domain with multiple tandem tetratricopeptide repeats and a NatB_MDM20 non-catalytic domain (first identified in yeast Mdm20p [23]). TPR domains are composed of 3-16 degenerate tandem repeats of 34 amino acids that form structural domains in proteins that facilitate the assembly of large protein complexes (reviewed in [45]). The NatB_MDM20 domain, of unknown function, is very conserved and occupies half of the NatB auxiliary subunit in all organisms studied. TCU2 shows 18.70% identity and 40.30% similarity to Mdm20p. Overall similarity and identity percentages between TCU2 and several of its putative orthologues in higher plants were of 71.02 and 59.81 (castor oil plant), 72.45 and 61.04 (grapevine), 62.47 and 49.77 (rice), and 69.74 and 59.80 (poplar), respectively (Figure S5).

The two mutant mRNA variants produced by the *tcu2-1* allele are translated into truncated proteins of 434 and 436 aa (Figure 4), which lack most of the NatB_MDM20 domain but still contain an intact protein-binding TPR domain. The NatB_MDM20 domain affected by the *tcu2-1* mutation is highly conserved in both monocotyledoneous and dicotyledoneous plants. The *tcu2-2* allele carries a T-DNA insertion in exon seventeen, disrupting the region that encodes the NatB_MDM20 domain (Figure 4).

### Expression of the *TCU2* gene and subcellular localization of the TCU2 protein

We determined the expression pattern of *TCU2 in silico*, according to the LRU Arabidopsis database Browser (http://www.bar.utoronto.ca/efp/cgi-bin/efpWeb.Cgi; http://www. genevestigator.ethz.ch; [46]) (Figure S6). To experimentally assess the spatial expression pattern of *TCU2*, we generated a *TCU2_pro_:GUS* construct (see Methods), in which the *TCU2* endogenous promoter drives expression of the β-glucuronidase (GUS; [47]) gene, and transferred this construct into plants. GUS activity was detected in all stages and tissues analyzed (Figure S7), and expression of the reporter gene was higher in the vascular bundles, hydathodes, leaf primordia and the base of the trichomes.

To assess the subcellular localization of the TCU2 protein, we generated a *35S_pro_:TCU2:GFP* construct (see Methods) and transferred it into plants. The GFP fluorescent signal was concentrated in the cytoplasm of proliferating cells and absent from the nucleus (Figure S8).

### Silencing of the *TCU2* gene

**Figure 7:**
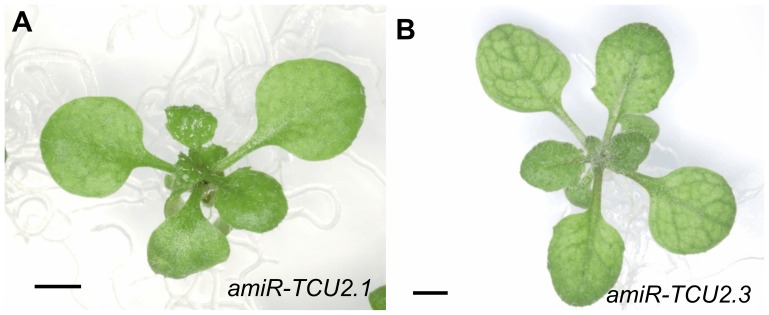
Rosette phenotype of *amiR-TCU2* transgenic lines. Pictures were taken 16 das. Scale bars: 2 mm.

To further confirm the effects of *TCU2* loss of function, we assessed the consequences of the post-transcriptional repression of this gene. A transgene designed to express amiR-TCU2, an artificial microRNA (amiRNA) targeting the At5g58450 gene, was constructed, cloned and transferred into Arabidopsis plants (see Methods). Among the 48 T_1_ transformants that we obtained, two showed in their T_2_ progeny the weak leaf folding shown by the *tcu2* mutants (Figure 7). These amiRNA-producing transgenic lines also exhibited reticulated leaves and mild early flowering, similar to *tcu2-1* and *tcu2-2* (*amiR-TCU2.1*, 22.87±1.46 das; *amiR-TCU2.3*, 25.26±0.96; Col-0, 27.87±1.70; n = 30).

### Double mutant combinations of *tcu2* alleles with *nbc-1* and *er-1*

At1g03150 encodes the putative catalytic subunit of Arabidopsis NatB. To identify phenotypes shared by mutants affected in the non-catalytic and catalytic subunits of the NatB complex, we obtained the only available line (SAIL_323_B05) carrying a T-DNA insertional allele of At1g03150, which is in the Col-0 genetic background. We named this allele *nbc-1* (for *NatB catalytic subunit-1*). It carries a T-DNA insertion that maps 25 bp upstream of nucleotide +1 of the transcription unit of At1g03150 and 85 bp upstream of nucleotide +1 of At1g03140, which are divergently transcribed.

Homozygous *nbc-1* plants exhibited a mutant phenotype distinguishable from both *tcu2-2* and Col-0. However, *tcu2-2* and *nbc-1* plants shared some traits, such as reticulated leaves, early flowering, increased proportion of siliques with more than two valves and aborted or unfertilized ovules in siliques (Figure S9). We also obtained *tcu2-2 nbc-1* double mutant plants, which exhibited an additive overall phenotype, except that the number of siliques per plant, scored 55 das, was strongly reduced in the double mutant: 235.00±24.00 siliques in Col-0, 250.20±52.67 in *tcu2-2*, 255.00±96.10 in *nbc-1*, and 154.5±33.49 in *tcu2-2 nbc-1*. More than two valves were seen in 0.60±0.89 siliques in Col-0, 7.20±9.63 in *tcu2-2*, 5.60±5.59 in *nbc-1*, and 5.60±7.30 in *tcu2-1 nbc-1*.

Measurement of plant height revealed that *tcu2-1*, *tcu2-2* and *nbc-1* genetically interact with *erecta-1* (*er-1*), the loss-of-function allele of the *ERECTA* gene carried by the L*er* accession [48]. Plant height measured at 49 das was 39.10±6.18 cm in L*er* (*er-1*), 50.28±4.89 cm in Col-0, 35.22±4.54 cm in *tcu2-2*, 7.05±2.23 cm in *tcu2-2 er-1*, 34.86±8.06 cm in *nbc-1*, and 15.75±7.08 cm in *nbc-1 er-1* (n = 10 plants). The interaction between *ER* and *TCU2* explains the difference in height between *tcu2-2 pnh-2* and P14 6.3 (*tcu2-1 zll-2 er-1*). The reduction in inflorescence size caused by the interaction between *tcu2* alleles and *er-1* causes a reduction in silique number (239.40±64.01 siliques per plant in L*er*, and 63.50±20.34 in P14 6.3; n = 5 plants; 59 das).

## Discussion

### *TCU2* is the Arabidopsis ortholog of the human and yeast genes encoding the NatB non-catalytic subunit

Although many eukaryotic proteins are subjected to N-α-terminal acetylation, the function of this co- and post-translational modification is known only for a few proteins in a few species, *Homo sapiens* and *Saccharomyces cerevisiae* in particular. Knowledge of this process in the plant kingdom is particularly poor: only a few genes encoding components of the N-α-terminal acetylation machinery have been studied, and only one of these genes, *ATMAK3*, has been found to cause a visible phenotype when mutated. *ATMAK3* encodes a cytoplasmic protein, the Arabidopsis ortholog of the catalytic subunit of human and yeast NatC complex; the *atmak3-1* mutant exhibits slightly pale leaves and reduced photosynthesis and growth [33].

We describe here an EMS-induced Arabidopsis mutant line that we initially named P14 6.3, which was found to carry the *tcu2-1* and *zll-2* point, loss-of-function alleles of the linked *TCU2* and *AGO10* genes, respectively. We positionally cloned *TCU2*, which encodes a protein homologous to the non-catalytic subunit of the NatB N-α-terminal acetylase complex of yeast and mammals, and identified *tcu2-2*, a T-DNA insertional allele of *TCU2*. We found that *TCU2* is broadly expressed in plants, and that TCU2 protein is present in the cytoplasm. These are the expected spatial and subcellular localization patterns for NatB-mediated N-α-terminal acetylation enzymatic activity, which is known to occur in all animal tissues in the cytoplasm (reviewed in [1]).

Loss of function mutation of *TCU2* causes a pleiotropic phenotype that includes leaf reticulation and weak leaf folding, early flowering, unfertilized or aborted ovules in siliques, and an increase in the proportion of siliques with more than two valves compared to wild type. These phenotypes were shown by homozygous *tcu2-1* or *tcu2-2* plants, the heterozygous F_1_ progeny of their intercrosses, and transgenic lines expressing an amiRNA targeting *TCU2*. These observations suggest that the non-catalytic subunit of the NatB N-α-terminal acetylase complex is required for N-α-terminal acetylation of proteins in Arabidopsis and that NatB-mediated N-α-terminal acetylation is required in Arabidopsis for vegetative and reproductive development, including leaf development, flowering time, flower and silique development, as well as gametogenesis and/or fertilization. These observations substantially expand the list of developmental processes for which N-α-terminal acetylation in general, and that mediated by NatB in particular, is known to be required in Arabidopsis.

We also studied the *nbc-1* mutant, which carries a loss-of-function allele of the gene encoding the putative catalytic subunit of NatB. The *tcu2* and *nbc-1* mutants showed reticulated leaves, early flowering, increased proportion of siliques with more than two valves, and aborted or unfertilized ovules in siliques. These shared traits clearly indicate that the NBC and TCU2 proteins participate in the same processes, as can be expected for the subunits of a complex. Further studies of the *nbc-1* and *tcu2* mutants will be required to explain the differences between their phenotypes and will provide useful insights into the N-α-terminal acetylome in plants.

### *TCU2* is required for vegetative and reproductive development

Several large-scale analyses of the N-α-acetylome have been recently conducted; these studies aimed to answer the many open questions on the functional role of Nat complexes in *Saccharomyces cerevisiae* [49], [50], *Homo sapiens* [35], [50]-[52], *Drosophila melanogaster* [53], *Arabidopsis thaliana* [35], *Halobacterium salinarum* and *Natronomonas pharaonis* [54], [55]. Although these studies provide a much better overview of the process at the proteome scale, gene-centric analyses are lacking, at least in the plant kingdom. Given that the TCU2 protein is highly conserved among higher plants, the *tcu2* alleles that we describe here will inform analyses of NatB function.

In the *tcu2* mutants, leaves are reticulated, a phenotype that is usually caused by defective mesophyll development [56]. Cross sections showed an increase in mesophyll intercellular air spaces. In previously described reticulated mutants [56]-[60], leaf interveinal tissues contain fewer and smaller mesophyll cells than wild type. Plastid number, size and/or differentiation status is also altered in reticulated mutants, suggesting a perturbation in retrograde signaling (from the chloroplasts to the cell nucleus), which in turn affects mesophyll growth and development [61]. A limited supply of essential metabolites in early stages of leaf development has also been proposed as a cause of the defective mesophyll structure seen in reticulated mutants: early differentiating tissues, as the perivascular, bundle-sheath cells, would be able to properly differentiate, but differentiation of the remaining tissues would be arrested or retarded [62]. Metabolic pathways that take place at least in part within chloroplasts are impaired in Arabidopsis reticulated mutants, such as *reticulata* (*re*), *venosa3 (ven3)* and *ven6*, and *chlorophyll a/b binding protein underexpressed (cue1). CUE1* encodes a phosphoenolpyruvate (PEP)/phosphate antiporter localized at the chloroplast inner membrane [63], and *VEN3* and *VEN6* encode the two subunits of carbamoyl phosphate synthetase (CPS), which is required in the chloroplasts for arginine biosynthesis [60].

Because of the known relationship between the reticulated phenotype and chloroplast dysfunction, a tempting hypothesis is that some processes related to chloroplast metabolism and/or biogenesis are impaired in the *tcu2* mutants. It is worth mentioning here that a recently discovered kingdom-specific property of the Arabidopsis N-α-acetylome is the high prevalence of N-α-terminal acetylation of neo N-termini of nuclear-encoded proteins imported into the chloroplast. These proteins are processed to remove their chloroplast transit peptides during translocation to the chloroplast and seem to be N-α-terminal acetylated after removal of the transit peptide. Nothing is known on the mechanism underlying such post-translational modification, which was shared by 277 peptides of a total of 1054 found to be N-α-terminal acetylated [35].

Only a few mutations have been reported to increase the number of Arabidopsis silique valves, most of them exhibiting low penetrance. Several of these mutations seem to perturb the regulation of floral meristem function through the *CLAVATA* (*CLV*)/*WUSCHEL* (*WUS*) pathway. Mutations in the *CLV* genes produce fruits with extra valves (reviewed in [64], [65]). Also, *FILAMENTOUS FLOWER* (*FIL*) and *YABBY3* (*YAB*3) negatively regulate *CLV3* and *WUS* [66], and many of the fruits of *fil yab3* double mutants have three valves [67]. The *CLV* genes encode regulatory peptides that are known to have different modifications, one of which might be N-α-terminal acetylation. In fact, the amino terminus of CLV3 is Met-Asp, one of the canonical NatB substrates.

### *TCU2* alleles synergistically interact with alleles of *AGO10* and *ER*

microRNAs (miRNAs) participate in the regulation of many aspects of plant development [68]-[70], including leaf [71], [72] and fruit [73] development. RNA-induced silencing complexes (RISC) mediate the action of miRNAs on their target mRNAs. A key RISC component in Arabidopsis is AGO1, and the closest AGO1 paralog in Arabidopsis is AGO10 [74]. AGO10 was proposed to participate in the miRNA pathway causing translational repression of miRNA targets [75] and has recently been found to regulate AGO1 by sequestering miRNAs that down-regulate members of the HOMEODOMAIN-LEUCINE ZIPPER (HD-ZIP III) family of transcription factors [40]. Reduction of mRNA levels of HD-ZIP III genes leads to defects in shoot apical meristem functions, alteration of organ polarity and abnormal formation of carpels. AGO10 participates in the repression of *APETALA2* (*AP2*), which promotes *WUS* activity [38].

We segregated the *tcu2-1* and *zll-2* mutations from the *tcu2-1 zll-2* double mutant. We also obtained *tcu2-2 pnh-2*, another combination of loss-of-function alleles of *TCU2* and *AGO10*. We considered some phenotypic traits of the *tcu2-1 zll-2* double mutant to be additive. For example, mutant alleles of *AGO10* cause fusion of the first two leaves, silique shortening and generalized formation of more than two valves in siliques; mutant alleles of *TCU2* cause leaf reticulation, early flowering and an increase in the proportion of siliques with more than two valves. As mentioned above, alteration in the number of silique valves is a rare phenotype; the observation of a perturbation in this trait in the *tcu2* and *ago10* mutants, though at different penetrance levels, cannot be put down to chance and strongly suggests that both *TCU2* and *AGO10* participate in fruit development.

Comparison of the phenotypes of the *tcu2-1*, *tcu2-2*, *zll-2* and *pnh-2* single mutants and their double mutant combinations clearly indicates that the *TCU2* and *AGO10* genes interact. The strong leaf folding shown by the double mutants is clearly a synergistic phenotype, which is absent from the single mutants. Indeed, leaves are almost normal in the *pnh-2* and *zll-2* mutants, and only mildly bent in the *tcu2* mutants. The leaf phenotype of the double mutants carrying mutant alleles of *TCU2* and *AGO10* is very similar to that of single mutants affected in *AGO7*, another member of the AGO family [76], when grown in our standard culture conditions (data not shown). Taken together, these observations suggest a functional relationship between *TCU2* and *AGO10*, in leaf development in particular. Two simple hypotheses may explain these observations: the AGO10 protein itself, or any of the proteins regulated by AGO10, is acetylated by NatB in the wild type, or the expression of *TCU2* is directly or indirectly regulated by the miRNA pathway. However, the N-terminus of the AGO10 protein does not show any of the canonical signals of NatB substrates, which makes unlikely its N-α-terminal acetylation by the NatB complex.

The genetic interactions that we found, of *er-1* with *tcu2* alleles and *nbc-1*, also indicate a functional relationship. The ER protein is assumed to be required for the regulation of plant size and belongs to a leucine-rich receptor-like serine/threonine kinase family of plant signaling receptors [48]. Further research will be required to shed light on the nature of the interactions of the *tcu2* and *nbc-1* mutations with *er-1*. These interactions, however, clearly suggest that L*er* is a better choice than other commonly used wild-type accessions for studies of mutations affecting components of Nat complexes, since it provides a sensitized genetic background that renders a much better visualization of the effects of loss of function of components of the Nat machinery.

## Materials and Methods

### Plant material and growth conditions

*Arabidopsis thaliana* (L) Heynh. (hereafter, Arabidopsis) Landsberg *erecta* (L*er*) and Columbia-0 (Col-0) wild-type accessions were obtained from the Nottingham Arabidopsis Stock Center (NASC; Nottingham, UK) and then propagated at our institute for further analysis. The P14 6.3 and P14 20.1 lines were isolated in a L*er* background after EMS mutagenesis in a screen in which mutants were given protocol numbers, as PN X.Y: PN indicates the corresponding parental group, X refers to the number of the plate where the mutant was isolated and Y is an ordinal assigned to each of the mutants found in a given plate [36]. Each parental group was the pooled M2 seed progeny of about 600 M1 plants exposed to EMS. The P14 6.3 and P14 20.1 M2 mutant lines were considered likely to carry identical mutation(s) because they were phenotypically indistinguishable and had been isolated among seeds belonging to the same parental group (P14).

All other Arabidopsis seeds used in this work were provided by NASC or the Arabidopsis Biological Resource Center (ABRC; Columbus, Ohio, USA). Seed sterilization and sowing, plant culture and crosses were performed as previously described [36], [77]. In brief, seeds were sown on plates containing MS agar medium (half-strength Murashige and Skoog salts, 0.7% plant agar [Duchefa], pH 5.7, and 1% sucrose) and stratified (4°C in the dark) for 48 h and then transferred to either Conviron TC16 or TC30 growth chambers set to our standard conditions (continuous light at approximately 75 µmol·m^-2^·s^-1^, 20±1°C, 60-70% relative humidity). When required, plants were transferred into pots containing a 2∶2∶1 mixture of perlite:vermiculite:sphagnum moss and grown in walk-in growth chambers set to our standard conditions.

### Plant gross morphology, histology and histochemical assays

Leaf clearing and fixation, embedding, microscopy and morphometry were performed as previously described [58], [60], [78], [79]. Venation diagrams were obtained from micrographs by hand drawing on the screen of a Wacom Cintiq 18SX Interactive pen display (http://www.wacom.com/) and using the Adobe Photoshop CS3 (http://www.adobe.com) software. Morphometric analyses of the diagrams (n≥10) were performed with ImageJ 1.36b [80] (http://rsb.info.nih.gov/ij/index.html/), Scion Image 4.0.3.2 (http://scion-image.software.informer.com/) and NIS-Elements AR 2.30 (Nikon Imaging; http://www.nis-elements.com/). GUS assays were performed as previously described [81].

### Positional cloning and molecular characterization of *TCU2* and its mutant alleles

Low-resolution mapping of the *tcu2-1* mutation was performed as previously described [82], [83]. For the fine mapping of the *TCU2* gene, SSLP, SNP and In/Del markers were developed based on the polymorphisms between L*er* and Col-0 described in the Monsanto Arabidopsis Polymorphism Collection database (http://www.arabidopsis.org). Synthetic oligonucleotides were purchased from Sigma-Aldrich UK (Table S1). Genomic DNA was extracted, PCR amplified and sequenced as previously described [84]. For the sequencing of *TCU2* and *AGO10* alleles, PCR amplification products spanning their transcription units were obtained using wild-type and mutant genomic DNA as templates. To confirm the presence and position of T-DNA insertions, DNA was extracted and PCR amplified. Sequencing reactions, RNA extractions and qRT-PCR were performed as described in [85], using the primers shown in Table S2. The multiple alignment of protein sequences shown in Figure S6 was obtained using ClustalX 2.0 [86] and shaded with Boxshade 3.21 (http://www.ch.embnet.org/software/BOX_form.html).

### Next-generation sequencing

Total DNA was purified from P14 6.3 rosettes collected 18 das using the DNeasy Plant Midi Kit (Qiagen) following the instructions of the manufacturer and sent for sequencing at BGI-Hong Kong. Whole genome sequencing was performed in an Illumina HiSeq 2000 and returned paired reads with an average length of 90 nt. A reference-guided assembly of the clean reads and a SNP report were obtained with SeqMan NGen (Lasergene 11; DNASTAR), using as a reference the genome sequence of L*er*-0 obtained in the ‘19 genomes of *Arabidopsis thaliana*’ project, available at http://mus.well.ox.ac.uk/19genomes/.

### DNA constructs

Gateway (Invitrogen) entry and destination vectors were used to obtain all the constructs used in this work. To visualize *TCU2* expression, we PCR amplified a 1,349-bp segment of genomic DNA from L*er*, including most of the first exon of *TCU2* (At5g58440), the intergenic region between *TCU2* and At5g58450 and 324 bp from the 5′ end of the transcription unit of At5g58450. To visualize the subcellular localization of the TCU2 protein, we PCR amplified a L*er* genomic segment of 5.2 kb encompassing the entire *TCU2* transcription unit. The amplification products were cloned into pGEM-T Easy221, sequence verified and subsequently subcloned into pMDC164 (*TCU2_pro_:GUS*) or pMDC85 (*35S_pro_:TCU2:GFP*). The constructs obtained in this way were then transferred from *Escherichia coli* One Shot Match1 cells to *Agrobacterium tumefaciens* LBA4404, and the transformants thus obtained were used to infect Col-0 and L*er* plants by the floral dip method [87]. The transgenic plants were selected on MS agar medium supplemented with 15 µg·ml^-1^ hygromicin.

### amiRNA construction

Arabidopsis transgenic lines expressing the amiR-TCU2 artificial microRNA (5′- UAGGAGAUUACUUAAGUCGAC -3′) were obtained as will be described in detail elsewhere (Jover-Gil, Paz-Ares, Micol and Ponce, unpublished). In brief, the transgene designed to express amiR-TCU2 was constructed in the backbone of the gene encoding miR319a, an endogenous Arabidopsis miRNA. The sequence of the mature miR319a miRNA was replaced by that of amiR-TCU2, as described in [88] and at http://wmd3.weigelworld.org/cgi-bin/webapp.cgi. The amiR-TCU2 construct was flanked with attB1 and attB2 sites to enable use of the Gateway technology, and inserted by means of a BP reaction into pGEM-T Easy221. The plasmid obtained in this way was used to transform *Escherichia coli* DH5α cells. Plasmid DNA was isolated from transformants and the insert was transferred into the pMDC32 destination vector [89] and then mobilized into *Agrobacterium tumefaciens* C58C1 cells. Arabidopsis plants were transformed by infection using the floral dip method [87].

## Supporting Information

Figure S1 Leaf venation pattern in the P14 6.3 line. Diagrams are shown for first- and third-node leaves, indicating the leaf margin in orange. Duplicated primary veins are highlighted with arrows in the diagram representing a fused first pair of leaves in P14 6.3. Pictures were taken 21 das. Scale bars: 2 mm.
(PDF)

Figure S2 Fine mapping of the *TCU2* gene. Red bars represent segments of chromosome 5, and green boxes, BAC clones corresponding to the candidate interval, which is highlighted in blue. The number of informative recombinants identified for each of the molecular markers used for linkage analysis is shown in parentheses.
(PDF)

Figure S3 Some phenotypic traits of the *pnh-2* mutant grown under our standard culture conditions. (A) Apical region of the inflorescence. (B) Fused first pair of leaves (arrow). (C) Adult plant. (D) Flowers of the apical region of the inflorescence, some of them showing siliques beginning to elongate. (E) Mature siliques. (F-H) Detail of three-valved siliques: (F) closed and (G, H) longitudinally open through a septum. Arrows highlight a few aborted or unfertilized ovules. Pictures were taken (B) 18 das and (A, C, D-H) 50 das. Scale bars: (A, B, D, E) 2 mm, (C) 2 cm and (F-H) 1 mm.
(PDF)

Figure S4 Early flowering in the P14 6.3 line. (A) Lateral view of P14 6.3 and L*er* rosettes. (B, C) Cotyledons and vegetative leaves of (B) P14 6.3 and L*er* (C) arranged from left to right in order of appearance. Leaf reticulation is clearly visible in B. Pictures were taken (A) 25 and (B, C) 28 das. Scale bars: (A) 1 cm and (B, C) 2 mm.
(PDF)

Figure S5 Multiple alignment of the NatB_MDM20 domains of TCU2 and some of its putative orthologs in higher plants. At, *Arabidopsis thaliana* (NP_200653.2); Rc, *Ricinus communis* (XP_002516347.1); Vv, *Vitis vinifera* (XP_002273069.1); Pt, *Populus balsamifera* subsp. *trichocarpa* (XP_002319956.1); and Os, *Oryza sativa* (Os NP_001055256.1). A triangle indicates the conserved amino acid changed by the *tcu2-1* mutation. Amino acid residues identical or similar in all five sequences are shaded black or grey, respectively. See http://pfam.sanger.ac.uk/family/PF09797?type=Family#tabview=tab1 for a list of all the unique domain organisations or architectures in which this domain is found.
(PDF)

Figure S6 *TCU2* expression in Arabidopsis development. Expression data from the Arabidopsis Electronic Fluorescent Pictograph (eFP) Browser for At5g58450 (*TCU2*) expression levels throughout all Arabidopsis developmental stages.
(PDF)

Figure S7 Spatial expression analysis of *TCU2*. GUS staining of *TCU2_pro_:GUS* transgenic plants in (A) leaves, (B) a root, (C) the basal region of trichomes and (D-I) whole rosettes. Plant material was collected at the time shown in each picture (in das). Scale bars: (A) 1 mm, (B) 0.5 mm, (C) 100 µm and (D-I) 2 mm.
(PDF)

Figure S8 Subcellular localization of the TCU2 protein. Confocal micrographs are shown from the apex and root elongation zone of transgenic plants obtained after transformation by infection with *Agrobacterium tumefaciens* cells carrying the pMDC85 vector (A) without insert or (B) with the *TCU2* insert. GFP emission is shown in green. The cloning site of pMDC85 is flanked by two tandem 35S promoters and the GFP coding sequence. Hence, GFP is expressed from a *35S_pro_:GFP* transgene in the plant shown in A, and a TCU2:GFP fusion protein is expressed from a *35S_pro_:TCU2:GFP* transgene in the plant shown in B. Nuclear exclusion of the TCU2:GFP protein is clearly visible in the dividing cells of the root tip shown in B. Pictures were taken 14 das. Scale bar: 50 µm.
(PDF)

Figure S9 Effects of the *tcu2* and *nbc-1* mutations on embryonic development. Dissected siliques are shown from selfed (A) L*er*, (B) *tcu2-1*, (C) Col-0, (D) *tcu2-2* and (E) *nbc-1* plants. Arrows indicate abnormal seeds that are likely to be aborted or unfertilized ovules. Pictures were taken 59 das. Scale bar: 250 µm.
(PDF)

Table S1 Oligonucleotide sets used for the fine mapping of *TCU2*. *Labeled with HEX (4,7,2′,4′,5′,7′-hexachloro-6-fluorescein).
(PDF)

Table S2 Other oligonucleotide sets used in this work. ^a,b^These oligonucleotides include at their 5′ ends the ^a^attB1 and ^b^attB2 sequences, which are shown in italics.
(PDF)
