# Supplementary figures and images for: Mutation of an Arabidopsis NatB N-Alpha-Terminal Acetylation Complex Component Causes Pleiotropic Developmental Defects
Source: PLoS One. 2013 Nov 14;8(11):e80697. doi: 10.1371/journal.pone.0080697 (PMC3828409; doi:10.1371/journal.pone.0080697)

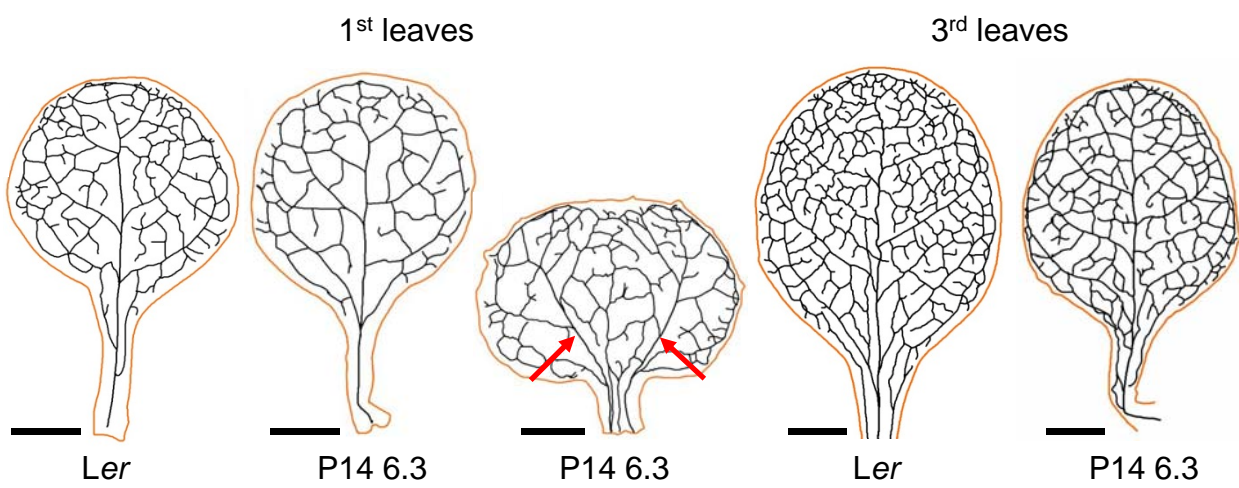

Supplement: Figure S1 — Leaf venation pattern in the P14 6.3 line. Diagrams are shown for first- and third-node leaves, indicating the leaf margin in orange. Duplicated primary veins are highlighted with arrows in the diagram representing a fused first pair of leaves in P14 6.3. Pictures were taken 21 das. Scale bars: 2 mm. (PDF) [file pone.0080697.s001.pdf]

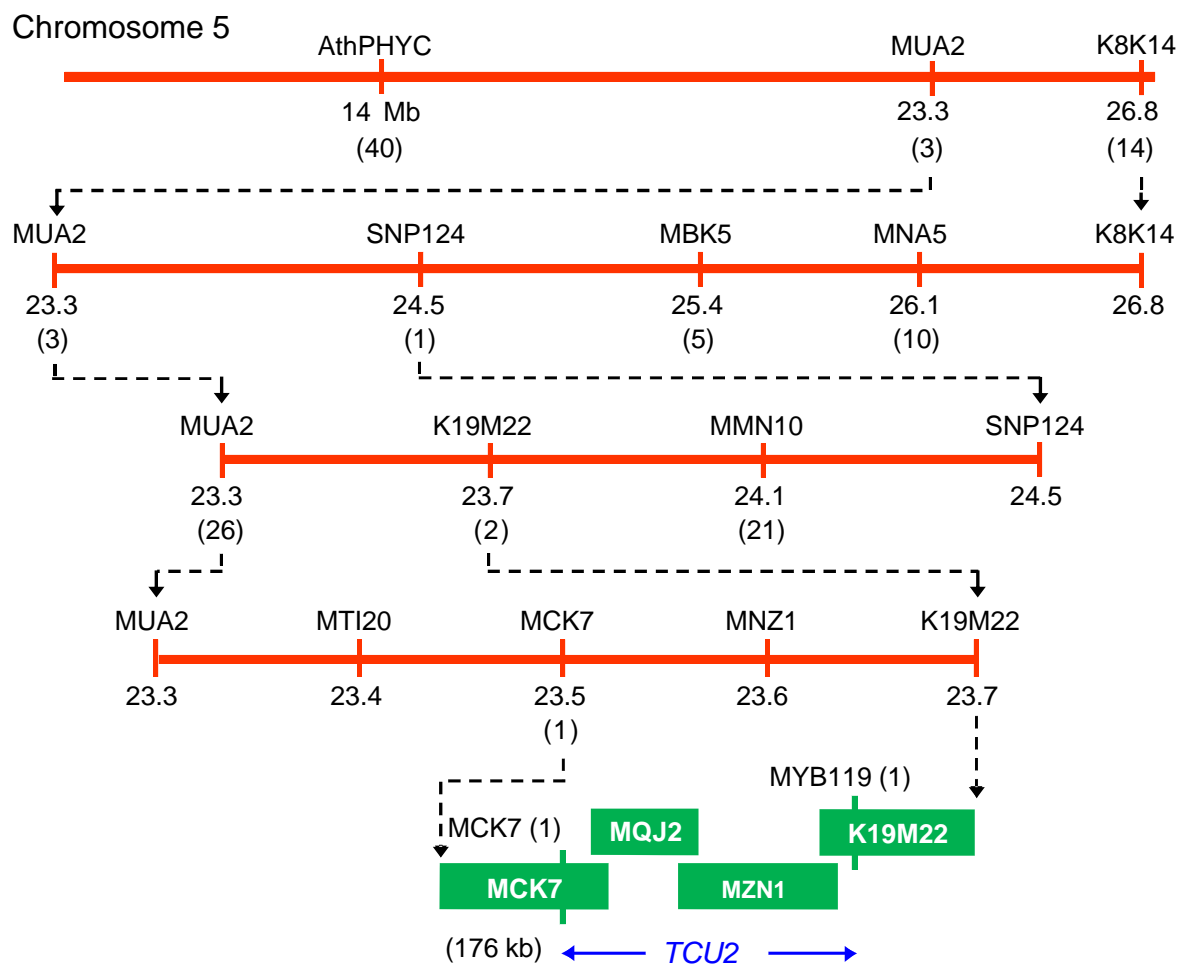

Supplement: Figure S2 — Fine mapping of the TCU2 gene. Red bars represent segments of chromosome 5, and green boxes, BAC clones corresponding to the candidate interval, which is highlighted in blue. The number of informative recombinants identified for each of the molecular markers used for linkage analysis is shown in parentheses. (PDF) [file pone.0080697.s002.pdf]

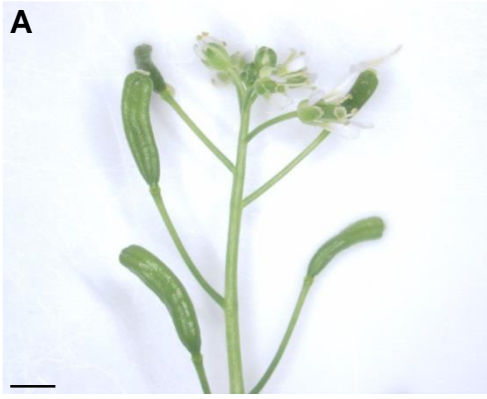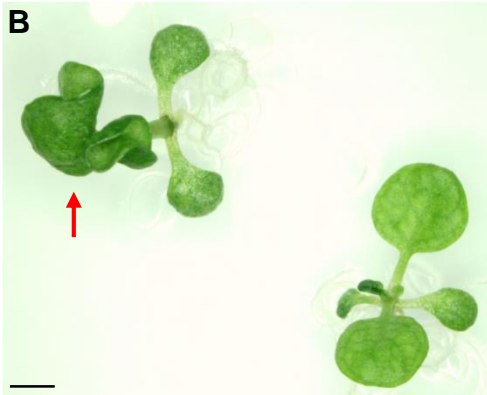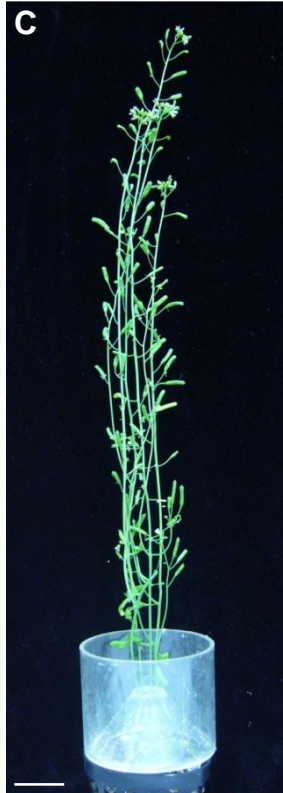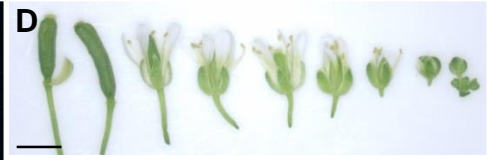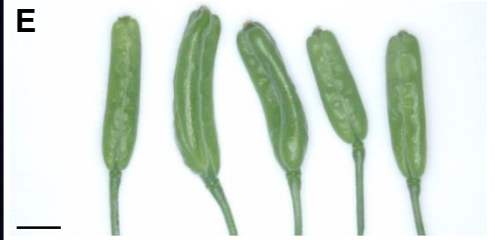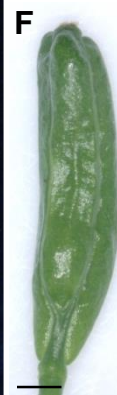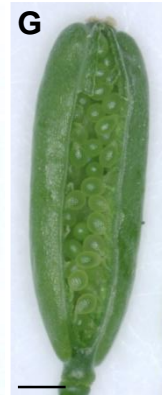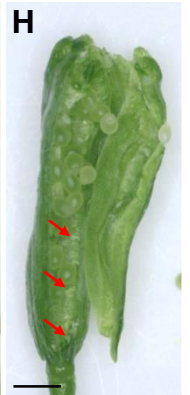

Supplement: Figure S3 — Some phenotypic traits of the pnh-2 mutant grown under our standard culture conditions. (A) Apical region of the inflorescence. (B) Fused first pair of leaves (arrow). (C) Adult plant. (D) Flowers of the apical region of the inflorescence, some of them showing siliques beginning to elongate. (E) Mature siliques. (F-H) Detail of three-valved siliques: (F) closed and (G, H) longitudinally open through a septum. Arrows highlight a few aborted or unfertilized ovules. Pictures were taken (B) 18 das and (A, C, D-H) 50 das. Scale bars: (A, B, D, E) 2 mm, (C) 2 cm and (F-H) 1 mm. (PDF) [file pone.0080697.s003.pdf]

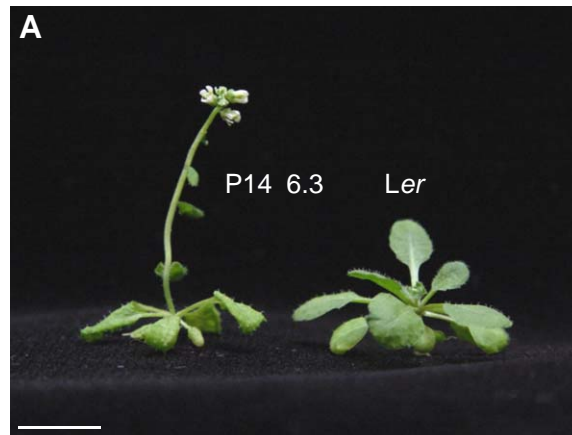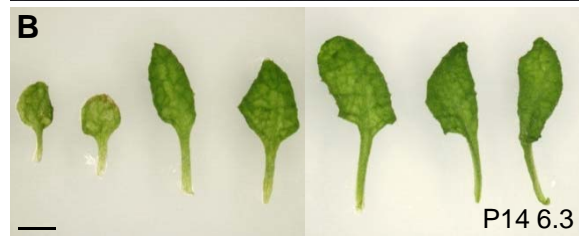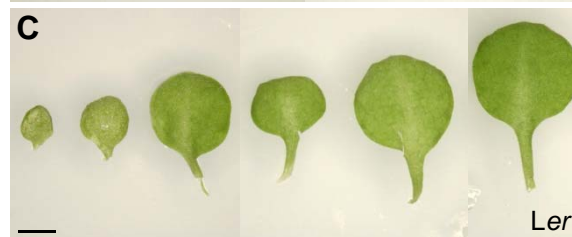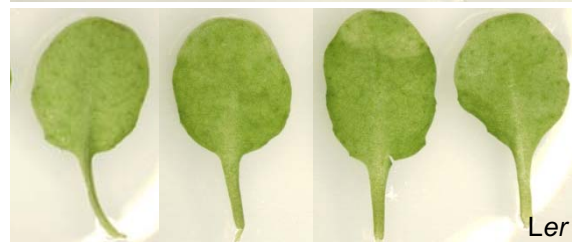

Supplement: Figure S4 — Early flowering in the P14 6.3 line. (A) Lateral view of P14 6.3 and Ler rosettes. (B, C) Cotyledons and vegetative leaves of (B) P14 6.3 and Ler (C) arranged from left to right in order of appearance. Leaf reticulation is clearly visible in B. Pictures were taken (A) 25 and (B, C) 28 das. Scale bars: (A) 1 cm and (B, C) 2 mm. (PDF) [file pone.0080697.s004.pdf]

At5g58450 247823\_at

Arabidopsis eFP Browser at bar.utoronto.ca  
Winter et al., 2007. PLoS One 2(8): e718

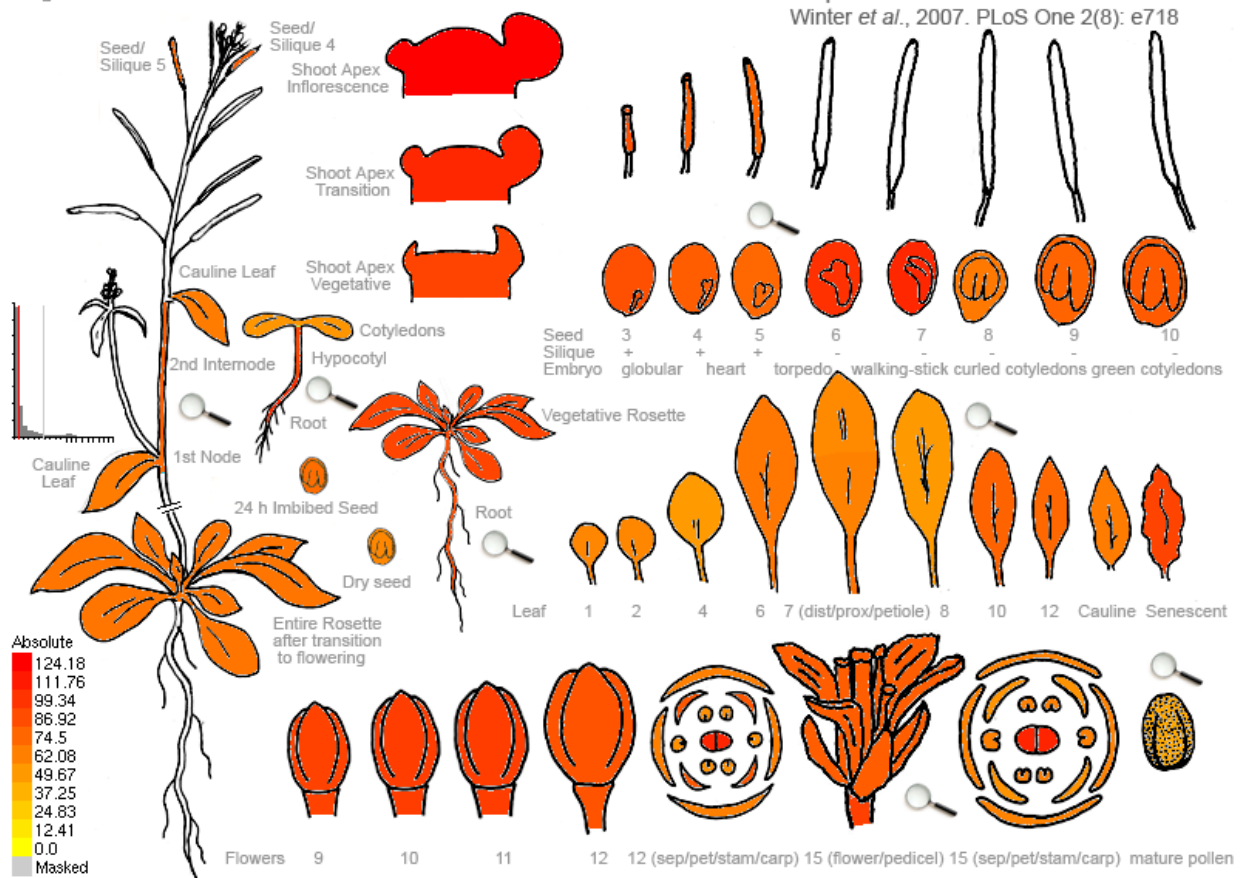

Supplement: Figure S6 — TCU2 expression in Arabidopsis development. Expression data from the Arabidopsis Electronic Fluorescent Pictograph (eFP) Browser for At5g58450 (TCU2) expression levels throughout all Arabidopsis developmental stages. (PDF) [file pone.0080697.s006.pdf]

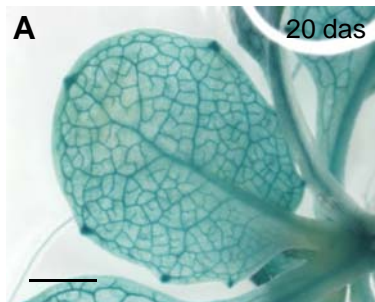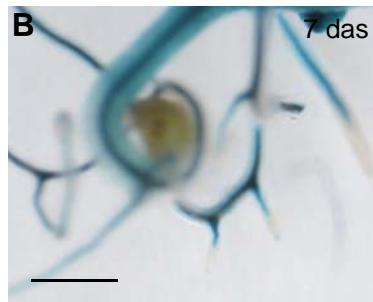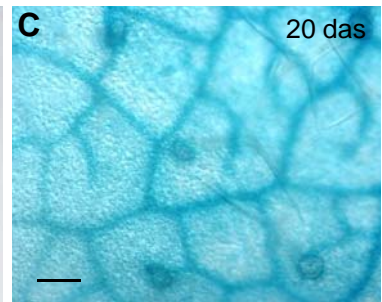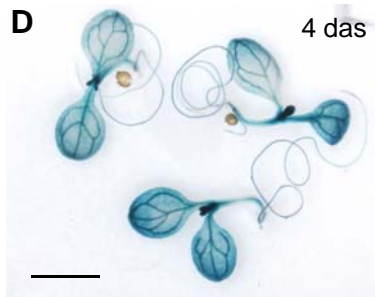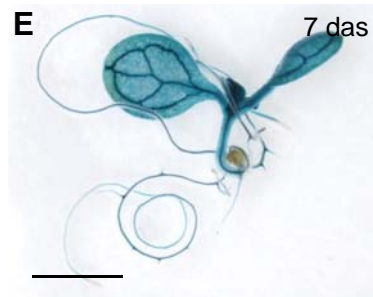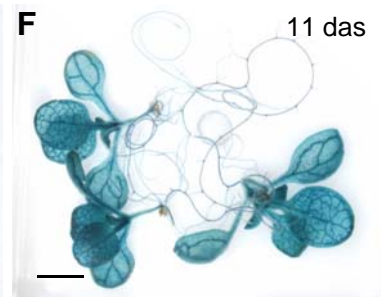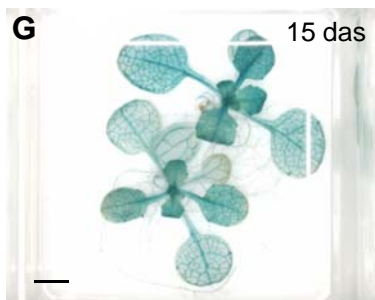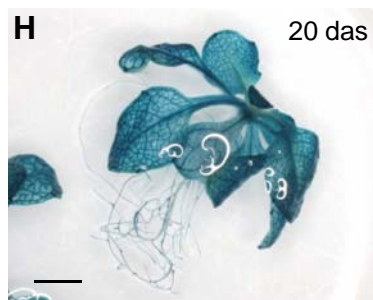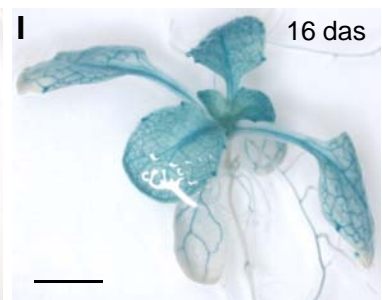

Supplement: Figure S7 — Spatial expression analysis of TCU2. GUS staining of TCU2pro:GUS transgenic plants in (A) leaves, (B) a root, (C) the basal region of trichomes and (D-I) whole rosettes. Plant material was collected at the time shown in each picture (in das). Scale bars: (A) 1 mm, (B) 0.5 mm, (C) 100 µm and (D-I) 2 mm. (PDF) [file pone.0080697.s007.pdf]

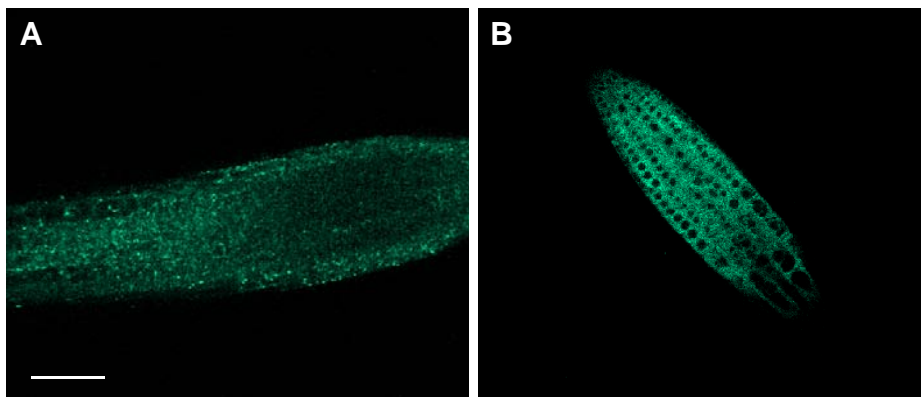

Supplement: Figure S8 — Subcellular localization of the TCU2 protein. Confocal micrographs are shown from the apex and root elongation zone of transgenic plants obtained after transformation by infection with Agrobacterium tumefaciens cells carrying the pMDC85 vector (A) without insert or (B) with the TCU2 insert. GFP emission is shown in green. The cloning site of pMDC85 is flanked by two tandem 35S promoters and the GFP coding sequence. Hence, GFP is expressed from a 35Spro:GFP transgene in the plant shown in A, and a TCU2:GFP fusion protein is expressed from a 35Spro:TCU2:GFP transgene in the plant shown in B. Nuclear exclusion of the TCU2:GFP protein is clearly visible in the dividing cells of the root tip shown in B. Pictures were taken 14 das. Scale bar: 50 µm. (PDF) [file pone.0080697.s008.pdf]

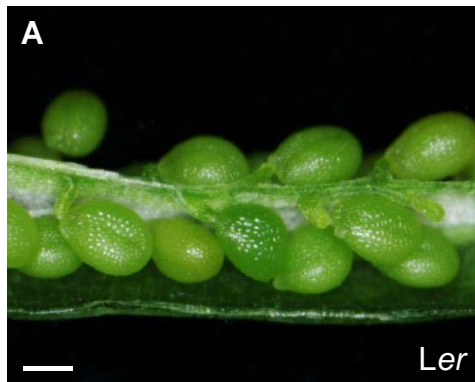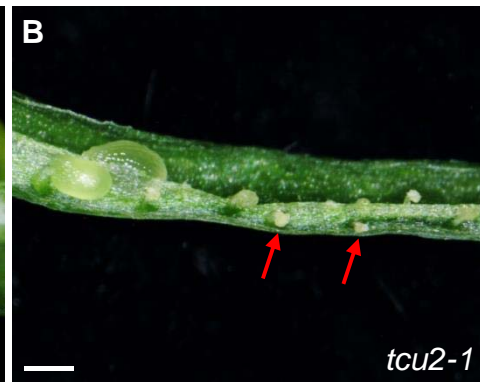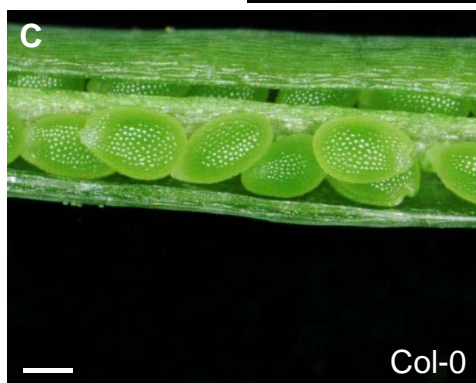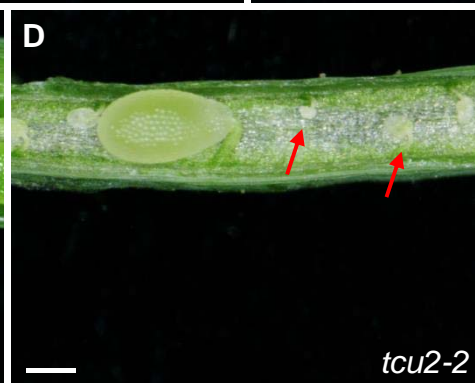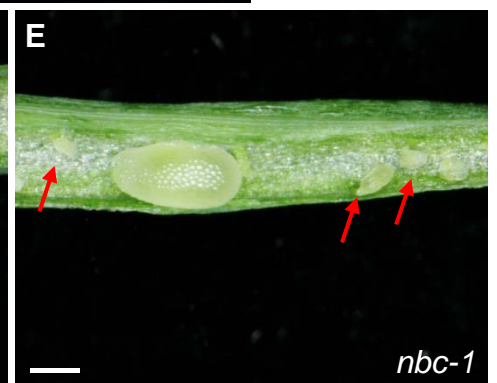

Supplement: Figure S9 — Effects of the tcu2 and nbc-1 mutations on embryonic development. Dissected siliques are shown from selfed (A) Ler, (B) tcu2-1, (C) Col-0, (D) tcu2-2 and (E) nbc-1 plants. Arrows indicate abnormal seeds that are likely to be aborted or unfertilized ovules. Pictures were taken 59 das. Scale bar: 250 µm. (PDF) [file pone.0080697.s009.pdf]
