# Supplementary material for: Mutation of an Arabidopsis NatB N-Alpha-Terminal Acetylation Complex Component Causes Pleiotropic Developmental Defects
Source: PLoS One. 2013 Nov 14;8(11):e80697. doi: 10.1371/journal.pone.0080697 (PMC3828409; doi:10.1371/journal.pone.0080697)
Supplement: Figure S5 — Multiple alignment of the NatB_MDM20 domains of TCU2 and some of its putative orthologs in higher plants. At, Arabidopsis thaliana (NP_200653.2); Rc, Ricinus communis (XP_002516347.1); Vv, Vitis vinifera (XP_002273069.1); Pt, Populus balsamifera subsp. trichocarpa (XP_002319956.1); and Os, Oryza sativa (Os NP_001055256.1). A triangle indicates the conserved amino acid changed by the tcu2-1 mutation. Amino acid residues identical or similar in all five sequences are shaded black or grey, respectively. See http://pfam.sanger.ac.uk/family/PF09797?type=Family#tabview=tab1 for a list of all the unique domain organisations or architectures in which this domain is found. (PDF) [file pone.0080697.s005.pdf]

```
At  365 …SHLTEEMFDSRISSASDLVQKLQRDAENSNLRGPYLAELEIEKRKFLFGKKNEDKLLES
Rc  319 …SHLADEVFDSRLSDASAFVQKLLADGNNGFIRSPYLAILEIERRRHLYGKANDDEIMEA
Vv  315 …SHLTDEVFISRLSNASAFAQKLQAEAGNDFIRCPYLANLEIERRKQLQGKGDDDKLIEV
Pt  307 …SQLADDVFHSRISTSLAFVKKLQADTSNDFIRCPYLATLEIERRKRLHGKGNDDDIVEA
Os  296 …TSLSDELVESRLASALSFVQKLQVNDTSDCVRGPHLASIEIERQRCRSGNPTDRKFIEA

At  424 LLQYFLKFGHLACYASDVEAYLQVLSPNKKAGFVEMLVKNSDSSASA-TKVLGQTTTILK
Rc  378 LLRYFYKFGHLACCTSDIEVFLQVLTPGKKMELVEKLVKSLDSLTTIPTKVLGQSITVFK
Vv  374 LMQYFFRFGHLACFASDIEGFLRVLPFGKKEEFLEKLIKSCDSLSAVPTKLLGQSISLFK
Pt  366 LMLYFLKFGHLASFSSDVEAFLQVLTPDKKTEFLAKLIKTLDSSASAPTKVLGQSITIFK
Os  355 LINYFHRFGHLSCAASDVEIYLHMLSSDETTELLDTISRSFD-ASSLSVKGLGLAITTFK
              ▲
            tcu2-1

At  484 VQELTGNIFGLPTDE-----------------------------IEASAVKLAKLYCQNL
Rc  438 IQQLIGNLYKLPVIG-----------------------------LEGFAKQMVEMYWKSL
Vv  434 IEELIGNMFKIPVVE-----------------------------LENSAIRMAQMYCKNL
Pt  426 IQELTGNMYKLPVLGACFFCFFCTHTLAHPTHNLLHIQTAQPLELEGCAVQMVEMYCKSL
Os  414 VQELLGTFFSKSTTE-----------------------------LQHIAKGMVEAFYKNL

At  515 SLSKDLDPQESMFGEELLSLISNMLVQLFWRTRDFGYLAEAIMVLELGLTIRGHVWQYKI
Rc  469 PLSKDLDPQESMHGEELLSMACNVLVQLFWLTRNVGYFMEAIMVLEFGLTIRPHVWQYKI
Vv  465 PLSKDLDQQESMHGEELLSMACNVLVQLFWRTRQLGYLLEAIMILELGLTIRRHVWQYKI
Pt  486 PLSKDLDPQESMHGEELLSMVCNVLVQLFWRTRHLGYFIEAIMVLEFGLTIRRYIWQYKI
Os  445 PLSRDLDPQESMHGEELLCMASSILVQLFWRTRNLGYLIEAILVLEFGLTVRKYVWQYKV

At  575 LLLHIYSYVGALPLAFERYKALDVKNILTETVSHHILRQMLESPMWVDLSNLLKDYLKFM
Rc  529 FLVHMYSHLGDLSLAYEWYKFLDVKNILMETVSHHIFPYMLPSPLWVDSSNLLKNYLRFM
Vv  525 LLVHLYSYLGAYSLSYEWYKSLEVKNILLESVSHHILPQMLVSPLWVDLNDVLKDYLKFM
Pt  546 LLLHLYSHLGAISLAYEWYKSLDVKNILMETVSHHILPQMLVSPLWGDLNNLLKDYLRFM
Os  505 MLVHLYSYLGALPLAHRWYVTLEVKNILLESASHHILPQMLNSPLLQQTADLVKDYLKFM

At  635 DDHLRESADLTFLAYRHRNYSKVIEFVLFKQRLQHSNQYQAARVEASVLQLKQNADSFEE
Rc  589 DDHFRESADLTFLAYRHRNYSKVIEFFQFKERLQQSNQYLVARVETSILQLKQKANNIEE
Vv  585 DDHLKESADLTSLAYRHRNYSKVIEFVQFKERLQHSNQYLMARLEAPILQLKLNANNIEE
Pt  606 DDHFRESADLTFLAYRHRNYSKVIEFVQFKERLQRSNQYLVARVETPILQLKQKADNIEE
Os  565 DDHLKESADLTCLAYRHRTYSKVIEFVQFKERLQHSMQYLSVRSDSIILSLKQKAESLDE

At  695 EERILENLKSGVQLVELSNEIGSRTLKFNEDM…
Rc  649 EEGILESLNCGSHFVELSNEIRSKSLTFNED-…
Vv  645 EECILESLKSRVHFPEFSSEIGGKSLTFNEDM…
Pt  666 EEGVLENLNGGVHFVELSNEIGSKNLTFNED-…
Os  625 VESILENVNHGARLVELSNEDNVKRFTFNEDL…
```
