# Supplementary material for: Mutation of an Arabidopsis NatB N-Alpha-Terminal Acetylation Complex Component Causes Pleiotropic Developmental Defects
Source: PLoS One. 2013 Nov 14;8(11):e80697. doi: 10.1371/journal.pone.0080697 (PMC3828409; doi:10.1371/journal.pone.0080697)
Supplement: Table S1 — Oligonucleotide sets used for the fine mapping of TCU2. *Labeled with HEX (4,7,2′,4′,5′,7′-hexachloro-6-fluorescein). (PDF) [file pone.0080697.s010.pdf]

Table S1.- Oligonucleotide sets used for the fine mapping of *TCU2*

Marker		Oligonucleotide sequences (5´→3´)		PCR product size (pb)	
Name	Type	Forward primer	Reverse primer	L*er*	Col-0
CER456965	In/Del	GGTTCTGTCTAGGTACACTGC	GTAAATGACACATATGGCGACAA	171	185
MTI20	In/Del	ACGTAATAAGCCCTTGCATTAAG	CCAGAGACAGTGGGGTAAGTC	194	177
K21L19	In/Del	TTCACTAATCCTAATTGGGGTAG	ATAGATCTCCGAACTCATCGTC	222	226
MCK7	In/Del	TTACACTTGTCCTCTCATTCGC	TTGGCTGATTGAAAGAAATAAAAC	176	202
MQJ2	In/Del	TGTTACACACCTTATGAGAACC	AAGAATATCGATCGGTGGTAAC	224	207
MZN1	In/Del	TTATATGAAACAGAGGAAATTTTCG	CATCATATATAACTAAACAATCTAG	181	185
K19M22	In/Del	TTACTTGCTGCTATGGTCTTCG	TGATTCAGCGATTGCAAGCGGC	174	164
MMN10	In/Del	CGCGGTAATAAGCTGCAATAAT	GATGTATGTTTCTCTGTCTCTG	337	405
MAF19	SNP	CGAATCAAATCGTCGGAGAGTA	ACACATGAATCAGAACAAATCACA	631	631
MBK5	SSLP	CTGTCAGTTGTTGGTGAAAG	TGAGCATTTCACAGAGACG*	360	381
MNA5	SSLP	TACAGAGAAAACATCTAACAGTC	AAAAGTCCCAACTCCAAATACC	168	142

*Labeled with HEX (4,7,2',4',5',7'-hexachloro-6-fluorescein).
