# Supplementary material for: Mutation of an Arabidopsis NatB N-Alpha-Terminal Acetylation Complex Component Causes Pleiotropic Developmental Defects
Source: PLoS One. 2013 Nov 14;8(11):e80697. doi: 10.1371/journal.pone.0080697 (PMC3828409; doi:10.1371/journal.pone.0080697)
Supplement: Table S2 — Other oligonucleotide sets used in this work. a,bThese oligonucleotides include at their 5′ ends the aattB1 and battB2 sequences, which are shown in italics. (PDF) [file pone.0080697.s011.pdf]

Table S2.- Other oligonucleotide sets used in this work

Purpose	Template	Oligonucleotide sequences (5'→ 3')	
Candidate gene sequencing	*AGO10*	TTCAAGATTAGCAAACAGTATCTG	CCACTAACAGTACCGCGAAC
	AGO10	AACAACAACATTTTTTACTTCACTG	CAGTGTAAAGACTTTTCCGGC
	AGO10	GTGTCATCAAAGAGTGTTAACAG	ACTGACTTCATAGTACAGTTCTC
	AGO10	GAAGGAAATACCGTGTTGCGGG	TTTCTCGACTTGATCGGGCC
	AGO10	TCATTTAGAAAAGCAACAGGGC	AAACATTGGTAAATCTAAAGCCG
	At5g58420	ACCCATAAATCCCAGAACCCG	ATGAAACCAGCAGGGTAGGTC
	At5g58420	CATCTGAAGAGGCTTAATGCG	TAGTAAAAAACAAGAGAGGACAC
	At5g58430	ATTTTCAAACAACGCGGCCAGG	ACTTTAAACAATCTCTCAGGCG
	At5g58430	GTTTCCTAGCGAACGTAGACTC	ACAATGACAAAAAAAGTGATCCAG
	At5g58440	CCATAGTCCCCACCTGCGC	TCCTTGAATAATCTCAGCAGATC
	At5g58440	GTGTTTCTTCAGGTGCAAGGG	TTGTCACCACCAAATACCTTCG
	At5g58440	GATGATGGCTGTCCAAGGCG	GGAAACAATGGGTATGAGGGC
	At5g58450	GTTGGAGAGTGTTGACATGGC	AATTCTTCTTTTATACGTGTGCAC
	At5g58450	GGAAAACAGATGAGGCACTCTC	GATATAGAAACAATCGAATGACC
	At5g58450	ACACAAACTCTCTCACTTTAAGC	CTTAGAAAGAGAAAGATTTTGGC
	At5g58450	GGCTTCCTACAGACGGTGTG	TCCTCAAAGCTGTCTGCGTTC
	At5g58450	CCATATCCCTGTGGGCAACC	CTGAACCAAGACCTGGACATC
	At5g58450	ACTGGCATGTGTTGAACTCCC	AGTGAGAAACCAGATGGGTGG
	At5g58460	ATCAATCATGGATCTATCGCC	AAACAAGCGGCCATGATAGC
	At5g58460	ATAGGAAAGTTCGCCATGTCAG	GTTTAAATGCGCTCTGCACTTG
	At5g58460	TCTGTATTCGCAATTCAGCTAG	CAAAACAATCCCCAATAATACAG
	At5g58465	CTTGTCTAACCGGTGACTAGC	TTACATGCAAATAATAAACTTACCC
	At5g58470	ATAAAGTTCCACTATTTTTGAGAG	TTCCCTCTGACTACCACTACC
	At5g58470	TAGAAACGATAGTGGTAGGAGC	CTCTGGTCAAAGGTAGGAGC
	At5g58470	TCTGCAGGCGGCTTTTTCAAC	ATATGGACGAGACCGGTTTCC

Table S2.- Other oligonucleotide sets used in this work

Purpose	Template	Oligonucleotide sequences (5'→ 3')	
Candidate gene sequencing	At5g58470	TAGAGGAGGAGGAGGTGGTG	CTTGGGATTTTCGGCATTAGTC
	At5g58480	ATAACAGTGAGATGCTGAGTCG	ATAATCATCTAACCTTTACGAAGG
	At5g58480	TTATGGAATGTGAATGAGTCGG	ATAATCATCTAACCTTTACGAAGG
	At5g58490	GAGGAGGAGGAGAAACAGCC	GTCTTCGACAGTGGATACCAC
	At5g58490	CCAAGTCCCAACTGGCCTGC	CAAACTTTAAAGAAAAATCTCAGAG
	At5g58495	CTATCCCAATTACAATGTCCCC	AAAAACAAAAAATAATAGTGGGTGG
	At5g58500	AACTTGTTGCTTCTGACTTGTG	CGTTAGACCCGAGACTTCGC
	At5g58530	CACTTTTTTATTAACCAGCCCTC	TAATGATTTGTTTTTTCTTTCGACG
	At5g58570	TCGGTTATGGGAGCCACACG	TTACATCGAGGATAGCGGGG
	At5g58600	CCTTCACATCTTTAGCAATGTTG	GTGAAAGACCAGAAATCAAACAC
	At5g58600	ATTGGTAATGAGGTTCGATTTCG	AGTTTCGACCCAATACGCCC
	At5g58600	CTTATTACCAAGACATGGACCG	CTTTTTTCCAATAAGCCCATCG
	At5g58650	CCGCCTTCTTAGCTGACACG	GCTGGAACCTTTCCGTGAAG
	At5g58670	CAACGGTTCTGTTTTCAGTCAG	AATGATCCCCCAAACGTCTTAC
	At5g58670	CTGCAGAAATGTCTTAACGTGG	ATCTAAAGGAAAGTCCATATTCC
	At5g58670	CTGCAGAAATGTCTTAACGTGG	GTCATACGTTTCATAATTTTGCTG
	At5g58680	TTCTCTCTTTCTCTGCAGTTGC	TGTCTTGTTAGTTCTATAGGG
	At5g58680	ATGATTGTTTCTTCCGGTGCGG	TGTCTTGTTAGTTCTATAGGG
	At5g58700	TAGTATGTTATTTATGGGGAGGG	CTTTGATCAAACTAAATTTAGGCC
	At5g58700	TAAAGATTGAGATTGAAAGATTACG	CAAATGTCTGAGTTATCATCTGC
	At5g58700	CAGTTTAAAGTTGCTAAGGTTTTC	TCTGCCATACCCCTACATTTAC
	At5g58700	ATTTCGTGTATCGTCATCCAGG	TAAGATGAGAGATGCTTATTAATG
	At5g58740	GTTAGAAACGGAGTGTACCTC	TCTTCTTGAAACCTCTGGAGC
	At5g58740	GCACATTACCCTGCAGAAGAG	CTCAGTGGACTTTTAGTAGATG
	At5g58750	GTTGCTTCGTTATGCTACATCG	GAATCAGAGCCATCTATGTAAC

Table S2.- Other oligonucleotide sets used in this work

Purpose	Template	Oligonucleotide sequences (5'$\rightarrow$ 3')	
Candidate gene sequencing	At5g58750	AGAAGATCACTCGTAGTTCCG	CATTCACATTTGTTCGGACGCG
	At5g58760	CGTGAATGGTGCTTCCGGCC	TGCACAATGAACTTGATCCGGG
	At5g58760	ACTCCAACCGAGAATTCCTCC	CTACTCGTTTGTGTGCAAGATC
	At5g58760	GAGCTTCTTCTCAGTTGTGGG	AAAAAAGAAGCAAAATTGCCGCC
	At5g58770	ATCATCTTCTTCCACACCGAC	CTCAACTTCGATTTCGCCATC
	At5g58770	ATCATCTTCTTCCACACCGAC	CCTGGTTTCGTATCAGCGCC
	At5g58780	TACAAGTTTAATACGTACGGCC	CTCAATTGAGTTGTACTTTATACC
	At5g58780	TACAAGTTTAATACGTACGGCC	ATTTGTTTTAGTGATAAGTAACGAC
	At5g58782	AAGGTTTCCAAAACTGCGAGAG	GATTATCTTTGTCCAGCTCAAAG
	At5g58784	AATACGATGATGAGGAAGAAAGG	TTTTGTGTTGATTGAGCCAGCC
	At5g58800	CATTTGTTTGACTAAATTATCCC	TAATGATGTAGAGGATAAATAGGG
	At5g58800	CAAAGAGTTTTTGTTGTCAGTAAC	TAATGATGTAGAGGATAAATAGGG
	FAS2	AAACCATATACGAACACTAATAAG	GATCCATCAGGTGACCATGAC
	FAS2	AGCTATGGTTGATAAATTCGGG	GATCCATCAGGTGACCATGAC
	FAS2	TATTTCTGGCTCAGTGGACAAC	GGTGTTGTTTCTGTCATTAGTTC
	FAS2	CGTCTTTCTTTCGAAGATTGTC	GTTAAAGATCAACACAACATCCC
GUS assays	*TCU2*	*GGGGACAAGTTTGTACAAAAAAGCAGGCT*CCCATCCA ACATCCGAGAGG[a]	*GGGGACCACTTTGTACAAGAAAGCTGGGT*ACATCAAAAG ACTCGGTTCCAG[b]
	pMDC164	AAGACTGTAACCACGCGTCTG	TTGACTGCCTCTTCGCTGTAC
Subcellular localization	*TCU2*	*GGGGACAAGTTTGTACAAAAAAGCAGGCT*ATGCGCA GGTGGGGACTATG[a]	*GGGGACCACTTTGTACAAGAAAGCTGGGT*AACATGGGAC ATCTGTTGCTTG[b]
	pMDC85	TTCATTTGGAGAGGACCTCG	TATGTTGCATCACCTTCACCCT

[a,b]These oligonucleotides include at their 5´ends the [a]attB1 and [b]attB2 sequences, which are shown in italics.
